# Ab Initio Ground-State Potential Energy Function and Vibration–Rotation Energy Levels of Magnesium Monohydride

**DOI:** 10.1021/acs.jpca.4c01757

**Published:** 2024-05-01

**Authors:** Jacek Koput

**Affiliations:** Department of Chemistry, Adam Mickiewicz University, 61-614 Poznań, Poland

## Abstract

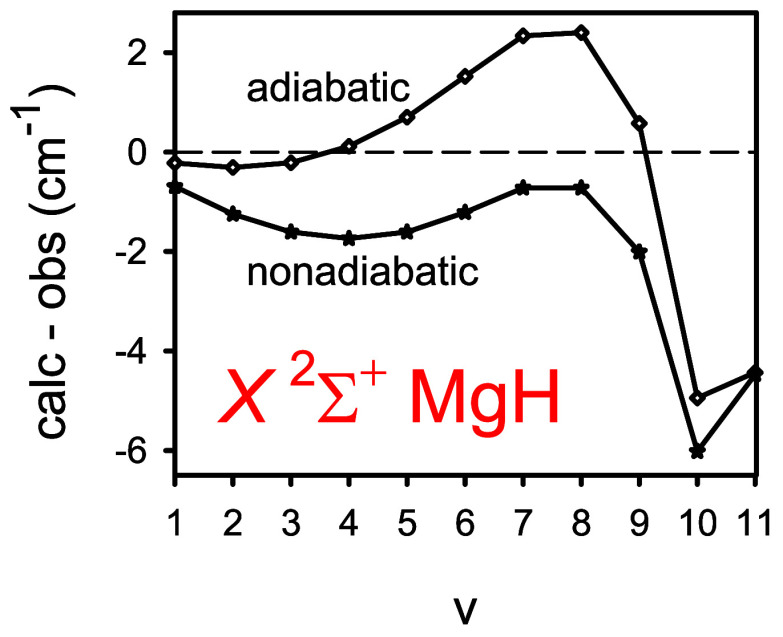

The accurate potential energy function of magnesium monohydride in its *X*^2^Σ^+^ state has been determined from ab initio calculations. The vibration–rotation energy levels of the main isotopologue ^24^MgH were predicted to near the “spectroscopic” accuracy. The scalar relativistic, adiabatic, and nonadiabatic effects were discussed.

## Introduction

Magnesium monohydride, MgH, is the only metal hydride known for which all of the vibration–rotation energy levels of the ground electronic state *X*^2^Σ^+^, up to the dissociation limit, were characterized by high-resolution spectroscopy. For perhaps the most complete review of numerous experimental spectroscopic studies on magnesium monohydride, the reader is referred to the recent work by Owens et al.^1^ The exhaustive experimental studies on the vibration–rotation energy levels of the isotopologues ^24^MgH and ^24^MgD were reported by Shayesteh et al.^2,3^ Those studies were further extended by Henderson et al.^4^ to include the species with magnesium minor isotopes ^25^Mg and ^26^Mg. The potential energy function of MgH in the *X*^2^Σ^+^ state was determined in a multi-isotopologue direct-potential-fit analysis. In particular, the binding energy *D*_e_ and equilibrium internuclear distance *r*_e_ of the main isotopologue ^24^MgH were derived to be 11,104.25 ± 0.8 cm^–1^ and 1.7296854 ± 0.0000007 Å, respectively.

The MgH radical was also the subject of numerous theoretical studies,^5−19^ and its electronic structure in the ground and excited electronic states was characterized at various levels of theory. In the most extensive studies to date,^15−19^ the potential energy functions for various electronic states of MgH were determined using the multireference configuration interaction (MRCI) method with correlation-consistent basis sets up to quintuple-zeta quality. Despite the high level of theory applied in those studies, the binding energy *D*_e_ and equilibrium internuclear distance *r*_e_ of MgH in the *X*^2^Σ^+^ state were found to span rather wide ranges 11,000–11,600 cm^–1^ and 1.729–1.744 Å, respectively. The experimental vibrational fundamental wavenumber ν of 1432 cm^–1^ (ref (2)) was also poorly reproduced, with the predicted values spanning the range of 1427–1438 cm^–1^. Given the progress in theoretical spectroscopy, such a situation is somewhat disappointing at present.

The aim of this work is to provide the accurate state-of-the-art potential energy function for the ground electronic state of MgH and to discuss the effects which should be taken into account in order to predict the vibration–rotation energy levels of MgH to near the “spectroscopic” accuracy.

## Method of Calculation

The molecular parameters of MgH in the *X*^2^Σ^+^ state were determined using the multireference averaged coupled-pair functional (MR-ACPF) method^20,21^ in conjunction with the augmented correlation-consistent core–valence basis sets up to octuple-zeta quality, aug-cc-pCV*n*Z (*n* = 5–8). The calculations consisted of two steps: the complete-active-space self-consistent-field (CASSCF) step, followed by the internally contracted^22^ MR-ACPF step. The usual full-valence active space, including the 3s- and 3p-like orbitals of the magnesium atom and the 1s-like orbital of the hydrogen atom, was extended with the 4s-, 4p-, and 3d-like orbitals of the magnesium atom. The CASSCF wave function of MgH included thus all excitations of three valence electrons in 14 molecular orbitals. In the vicinity of the equilibrium configuration of MgH, the occupancy of highest-energy active natural orbitals of the CASSCF wave function was about 0.0003*e*. In the generation of the CASSCF wave functions, the 1s-, 2s-, and 2p-like core orbitals of the magnesium atom were optimized, but they were kept doubly occupied. The reference function for the MR-ACPF step consisted of 263 configuration state functions. In this step, all but magnesium 1s electrons (11 electrons) were correlated with single and double excitations. The dynamical correlation effects of deep-core magnesium 1s electrons were thus neglected. The calculations were performed using the MOLPRO package of ab initio programs^23^ unless otherwise noted.

The core–valence basis sets cc-pCV*n*Z for magnesium were developed, up to quintuple-zeta quality, by Prascher et al.^24^ The larger basis sets of sextuple- through octuple-zeta quality were developed for the previous study on magnesium monohydroxide.^25^ For the sake of consistency, the quintuple-zeta quality basis set for magnesium was reoptimized. The cc-pCV5Z basis set for magnesium consists of a (20*s*, 14*p*, 8*d*, 6*f*, 4*g*, 2*h*) set contracted to a [11*s*, 10*p*, 8*d*, 6*f*, 4*g*, 2*h*] set. The cc-pCV6Z basis set consists of a (23*s*, 16*p*, 10*d*, 8*f*, 6*g*, 4*h*, 2*i*) set contracted to a [13*s*, 12*p*, 10*d*, 8*f*, 6*g*, 4*h*, 2*i*] set. The cc-pCV7Z basis set consists of a (26*s*, 18*p*, 12*d*, 10*f*, 8*g*, 6*h*, 4*i*, 2*k*) set contracted to a [15*s*, 14*p*, 12*d*, 10*f*, 8*g*, 6*h*, 4*i*, 2*k*] set. Also, the cc-pCV8Z basis set consists of a (29*s*, 20*p*, 14*d*, 12*f*, 10*g*, 8*h*, 6*i*, 4*k*, 2*l*) set contracted to a [17*s*, 16*p*, 14*d*, 12*f*, 10*g*, 8*h*, 6*i*, 4*k*, 2*l*] set. The diffuse functions (aug) were taken as the customary even-tempered functions with a multiplicative factor of 0.4. Further details of these new core–valence basis sets for magnesium will be reported elsewhere. The valence basis sets aug-cc-pV*n*Z for hydrogen were taken from the literature.^26,27^ Because the MOLPRO package cannot handle functions higher than *i*, the *k* and *l* polarization functions were omitted in the multireference calculations.

The vibration–rotation energy levels of magnesium monohydride were calculated using the Numerov–Cooley method.^28^ The energy levels were calculated using the nuclear masses of magnesium and hydrogen.

## Results and Discussion

**Figure 1:**
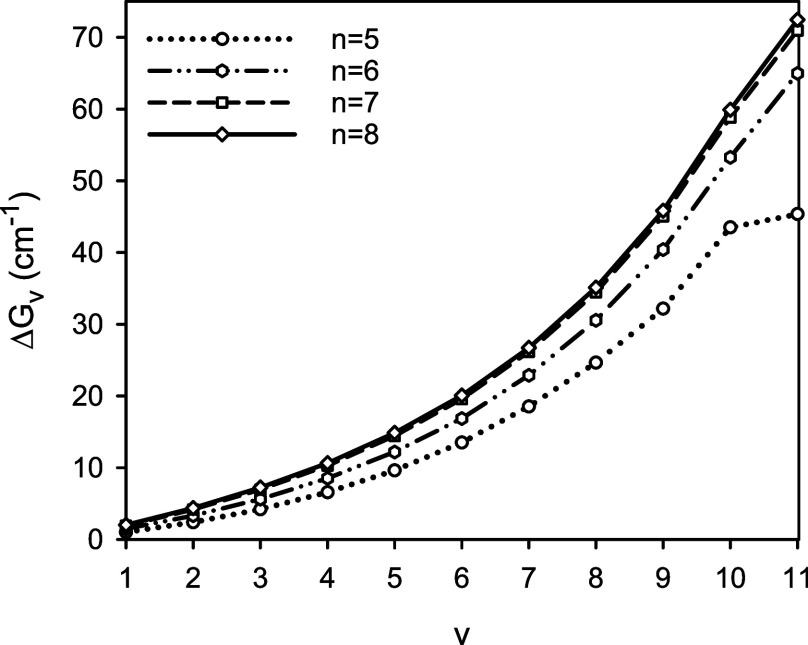
Differences between the calculated [MR-ACPF/aug-cc-pCV*n*Z (*n* = 5–8)] and experimental^3^ vibrational term values Δ*G*_v_ for the *X*^2^Σ^+^ state of ^24^MgH.

To determine the potential energy function of MgH in the *X*^2^Σ^+^ state, the total energies were calculated at the MR-ACPF/aug-cc-pCV*n*Z (*n* = 5–8) level of theory at 66 internuclear distances ranging from 0.9 to 50 Å. The vibration–rotation energy levels of the main isotopologue ^24^MgH were then calculated for the rotational quantum number *N* ranging from 0 to 3. The equilibrium internuclear distance *r*_e_ was determined by fitting the predicted total energies, in the close vicinity of the minimum, with a polynomial expansion. For a given vibrational state, the effective rotational constant *B*_v_ and quartic centrifugal distortion constant *D*_v_ were determined by fitting the predicted rotational energies with a power series in *N*(*N* + 1). The predicted molecular parameters are given in Table 1. The predicted values tend clearly to converge with enlargement of the one-particle basis set. The total energy at a minimum *E* is converged to better than 0.6 m*E*_h_. Concerning the basis set size, the best predicted binding energy *D*_e_ and the equilibrium distance *r*_e_ are estimated to be accurate to about ±2 cm^–1^ and ±0.0001 Å, respectively. The analogous error bars for the vibrational fundamental wavenumber ν and the effective ground-state rotational constant *B*_0_ are estimated to be about ±0.1 and ±0.0006 cm^–1^, respectively. Figure 1 illustrates the basis set convergence of the calculated vibrational term values *G*_v_. Differences between the calculated and experimental values Δ*G*_v_ are plotted for all of the observed^3^ bound vibrational levels of ^24^MgH, *v* = 1–11. For the highest-energy level, the calculated term values overestimate the experimental counterparts by as much as 45.4 through 72.5 cm^–1^ for the aug-cc-pCV5Z through aug-cc-pCV8Z(*i*) basis sets, respectively. Note that, somewhat surprisingly, the molecular parameters of MgH obtained with the largest basis set are in worst agreement with the experimental data. This suggests that some other effects should be considered, namely, enlargement of the basis set beyond the *i* polarization functions and of electron correlation beyond the MR-ACPF level of approximation, as well as the scalar relativistic, adiabatic, and nonadiabatic effects.

**Table 1:** Molecular Parameters for the *X*^2^Σ^+^ State of ^24^MgH Determined at the MR-ACPF/aug-cc-pCV*n*Z Level of Theory

	*n* = 5	*n* = 6	*n* = 7	*n* = 8
*r*_e_^a^ (Å)	1.73034	1.72977	1.72957	1.72948
*E* + 200^b^ (hartree)	–0.518218	–0.522530	–0.524054	–0.524613
*D*_e_^c^ (cm^–1^)	11,149	11,173	11,180	11,182
ν^d^(cm^–1^)	1432.96	1433.44	1433.87	1433.99
*B*_0_^e^ (cm^–1^)	5.73589	5.73942	5.74072	5.74128

a The equilibrium internuclear distance.

b The total energy at a minimum.

c The binding energy.

d The vibrational fundamental wavenumber.

e The ground-state effective rotational constant.

Effects of the *k* and *l* polarization functions were investigated using the partially spin-restricted coupled-cluster method RCCSD(T),^29^ as implemented in the OpenMolcas package of ab initio programs.^30^ In the vicinity of the equilibrium configuration of MgH, enlargement of the truncated basis set aug-cc-pCV8Z(*i*) to the complete basis set aug-cc-pCV8Z decreases the total energy by only 1.2 m*E*_h_. Changes in the molecular parameters of MgH listed in Table 1 were found to be also very small, being just about 0.000002 Å for *r*_e_, 1.4 cm^–1^ for *D*_e_, −0.01 cm^–1^ for ν, and 0.00003 cm^–1^ for *B*_0_.

Changes due to electron correlation beyond the MR-ACPF level of approximation were estimated in the full-configuration-interaction (FCI) calculations. The FCI calculations accounting for the correlation effects of all 11 outer-core and valence electrons of MgH appeared to be not feasible. Even the modest basis set of double-ζ quality was applied, the FCI wave function consisted of nearly 2 × 10^12^ determinants for each symmetry class. Thus, only three valence electrons of MgH were considered. In calculations with the aug-cc-pCVQZ basis set, differences between the FCI and MR-ACPF total energies of MgH were found to be smaller than 1 μ*E*_h_ for all of the internuclear distances under consideration. The valence CASSCF/MR-ACPF treatment based on the extended active space described above is thus close to the valence FCI treatment. However, note that in these correlation treatments, the core–valence and core–core correlation effects of eight outer-core electrons of magnesium were neglected. Changes in the total energy of MgH beyond the MR-ACPF level of approximation were considered negligible.

The scalar relativistic effects were investigated using the exact-2-component (X2C) approach.^31^ The scalar relativistic correction was determined as a difference in the total energy of MgH calculated at the MR-ACPF/aug-cc-pCV5Z(uncontracted) level of theory using either the X2C or nonrelativistic Hamiltonian. The extended active space was used, and all 11 outer-core and valence electrons of MgH were considered in the dynamical correlation treatment. In the vicinity of the equilibrium configuration of MgH, the scalar relativistic correction was determined to be −307.6 m*E*_h_.

The adiabatic effects were investigated for the main isotopologue ^24^MgH as well as for the minor isotopologues including ^25^Mg, ^26^Mg, D, and T. The diagonal Born–Oppenheimer correction (DBOC)^32,33^ was determined using the restricted-active-space configuration interaction (RAS CI)^34^ method with the aug-cc-pCVTZ basis set. The extended active space described above was taken as the active space (RAS2) of the RAS CI wave function of MgH. The calculations were performed using the PSI3 package of ab initio programs.^35^

**Figure 2:**
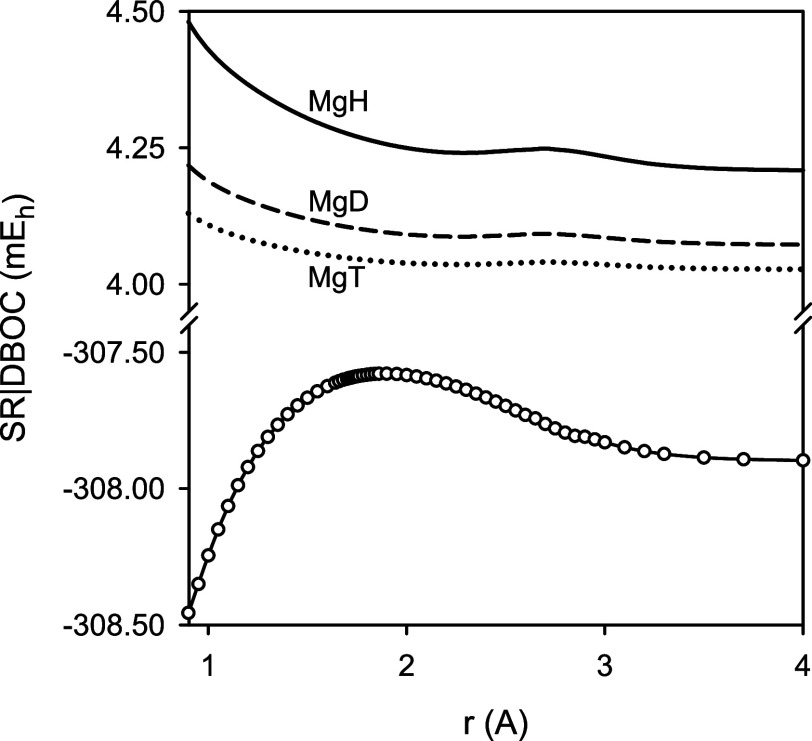
Predicted scalar relativistic (SR, the lower part) and diagonal Born–Oppenheimer corrections (DBOC, the upper part) to the total energy for the *X*^2^Σ^+^ state of MgH as a function of the internuclear distance *r*. Note that the upper-part energy scale is stretched by a factor of 2.

Figure 2 illustrates contributions to the total energy of MgH due to the scalar relativistic and adiabatic effects as functions of the internuclear distance *r*. The presented functions DBOC(*r*) were calculated for the ^24^MgH, ^24^MgD, and ^24^MgT isotopologues. In the vicinity of the equilibrium configuration of these isotopologues, the corresponding DBOC values were predicted to be 4.27, 4.10, and 4.05 m*E*_h_, respectively.

**Figure 3:**
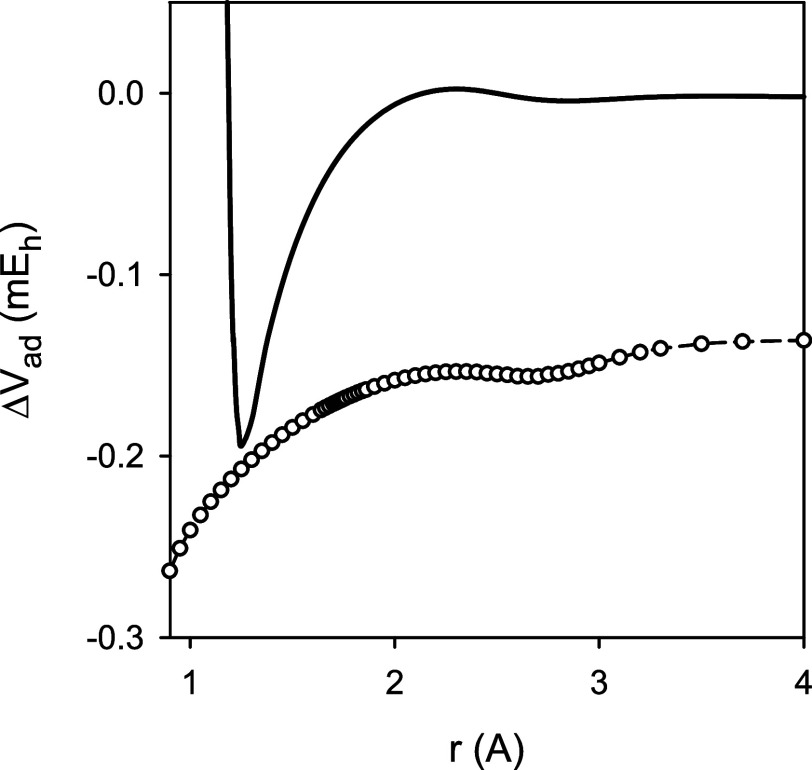
Experimental^4^ adiabatic correction function Δ*V*_ad_(*r*) for ^24^MgD vs ^24^MgH (solid line) in comparison to a difference of the corresponding diagonal Born–Oppenheimer corrections (circles) predicted in this work.

**Figure 4:**
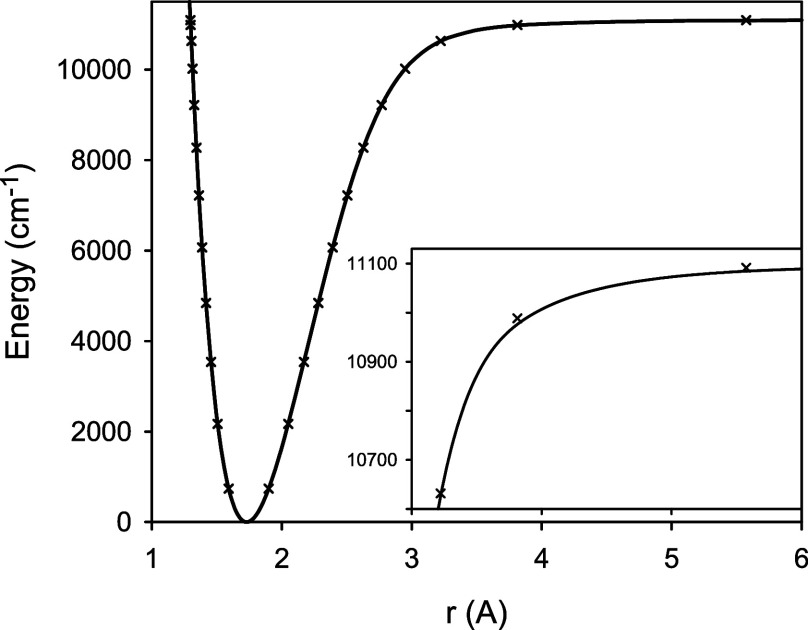
Predicted adiabatic potential energy function for the *X*^2^Σ^+^ state of ^24^MgH in comparison to the experimental^3^ classical turning points for the vibrational levels *v* = 0–11 (x marks). The inset shows the region of the outer turning points for the vibrational levels *v* = 9–11.

**Figure 5:**
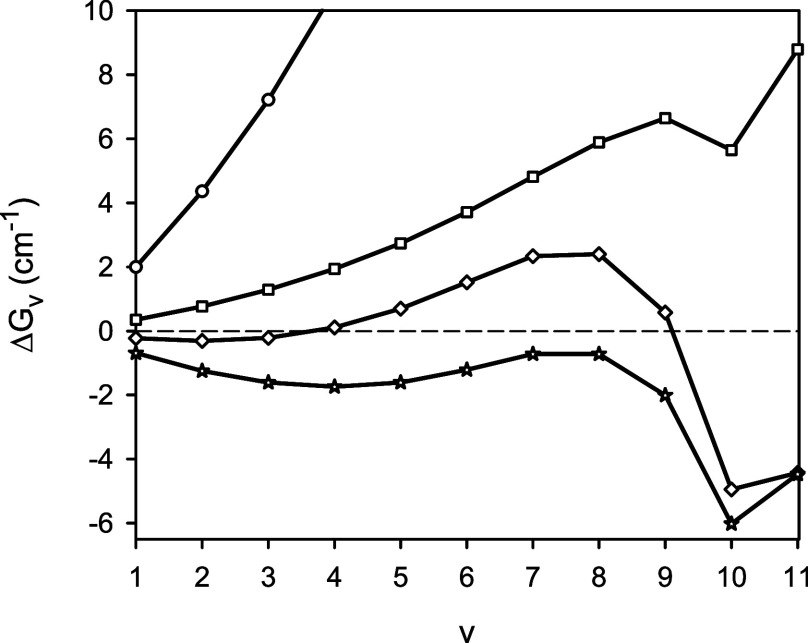
Differences between the calculated (see Table 2: CV circles, CV + R squares, and CV + R + D diamonds) and experimental^3^ vibrational term values Δ*G*_v_ for the *X*^2^Σ^+^ state of ^24^MgH. The plot “CV” extends far beyond the figure scale; compare Figure 1. The values marked by stars were predicted taking into account the nonadiabatic effects.

The energy corrections due to enlargement of the basis set and to the scalar relativistic and adiabatic effects were calculated at the same set of points as the MR-ACPF/aug-cc-pCV8Z(*i*) potential energy function discussed above. The molecular parameters for ^24^MgH in its *X*^2^Σ^+^ state obtained with the corrected potential energy functions are given in Table 2. These are compared with the corresponding values derived by Henderson et al.^4^ in the analysis of high-resolution vibration–rotation spectra of ^24^MgH and its minor isotopologues. The predicted adiabatic equilibrium internuclear distance *r*_e_ underestimates its experimental counterpart by 0.0004 Å. Accordingly, the predicted effective ground-state rotational constant *B*_0_ overestimates its experimental counterpart by 0.0057 cm^–1^. Somewhat surprisingly, this difference is another magnitude larger than the estimated uncertainty of the theoretical *B*_0_ value. For the ^24^MgH, ^24^MgD, and ^24^MgT isotopologues, the adiabatic equilibrium internuclear distance *r*_e_ is predicted to be longer than the Born–Oppenheimer counterpart by 0.00038, 0.00020, and 0.00014 Å, respectively. In the analysis of the experimental spectra of various MgH isotopologues,^4^ only a difference between the adiabatic potential energy functions Δ*V*_ad_ of ^24^MgD and ^24^MgH could be determined. Figure 3 illustrates the experimental difference Δ*V*_ad_ as a function of the internuclear distance *r*, compared with a difference of the corresponding diagonal Born–Oppenheimer corrections predicted in this work. Concerning the equilibrium internuclear distance *r*_e_, the experimental difference for ^24^MgD and ^24^MgH was derived^4^ to be −0.00024 Å, as compared to the theoretical value of −0.00018 Å. For the ^24^MgH, ^24^MgD, and ^24^MgT isotopologues, the adiabatic corrections to the Born–Oppenheimer binding energy *D*_e_ were predicted to be −14.1, −6.8, and −4.3 cm^–1^, respectively. From the experimental data,^4^ only the isotopic shifts in *D*_e_ could be derived. Relative to the main isotopologue ^24^MgH, the D-, T-, ^25^Mg-, and ^26^Mg-isotopic shifts in *D*_e_ were obtained^4^ to be 7.59, 10.11, −0.05, and −0.10 cm^–1^, respectively. The corresponding theoretical adiabatic values were predicted in this work to be 7.35, 9.80, −0.03, and −0.05 cm^–1^, respectively. The predicted adiabatic vibrational fundamental wavenumber ν and the binding energy *D*_e_ of ^24^MgH differ from their experimental counterparts by 0.2 and 3 cm^–1^, respectively. The predicted Born–Oppenheimer and adiabatic potential energy functions of ^24^MgH are given in Table S1 of the Supporting Information. The latter function is also shown in Figure 4 along with the superimposed experimental^3^ classical turning points for the vibrational levels *v* = 0–11. All of the experimental classical turning points coincide (in the scale of the figure) with the theoretical ones. Differences between the calculated and experimental vibrational term values Δ*G*_v_ of ^24^MgH are depicted in Figure 5. A comparison with Figure 1 shows that upon accounting for the scalar relativistic and adiabatic effects, these differences decrease by the order of magnitude. The calculated adiabatic vibrational term values *G*_v_ and the effective rotational constants *B*_v_ and quartic centrifugal distortion constants *D*_v_ of all bound vibrational states for the *X*^2^Σ^+^ state of ^24^MgH are given in Table 3. The predicted values are compared with the empirical band constants reported by Shayesteh et al.^3^ As shown in Table 3 and Figure 5, differences between the predicted and experimental spectroscopic constants of ^24^MgH change regularly with the increasing quantum number *v* up to *v* = 9. The analogous differences for *v* = 10 and 11 clearly do not follow that pattern.

**Table 2:** Molecular Parameters^a^ for the *X*^2^Σ^+^ State of ^24^MgH Determined using Various Potential Energy Functions and Derived from the Experimental Data

	CV^b^	CV + R^c^	CV + R + D^c^	Exp.^d^
*r*_e_ (Å)	1.72948	1.72890	1.72928	1.729685
*E* + 200 (hartree)	–0.525839	–0.833431	–0.829159	
*D*_e_ (cm^–1^)	11,183	11,115	11,101	11,104.3
ν (cm^–1^)	1433.98	1432.33	1431.76	1431.978
*B*_0_ (cm^–1^)	5.74130	5.74476	5.74223	5.736507

a See Table 1.

b The potential energy function was calculated at the MR-ACPF/aug-cc-pCV8Z level of theory, including the correction for enlargement of the basis set (see the text).

c Including additional corrections for the scalar relativistic (R) and adiabatic (D) effects.

d Derived from the experimental data in ref (4).

**Table 3:** Adiabatic Vibrational Term Values (*G*_v_) and the Effective Rotational (*B*_v_) and Quartic Centrifugal Distortion (*D*_v_) Constants (All in Inverse Centimeters) for the *X*^2^Σ^+^ State of ^24^MgH

	*G*_v_			*B*_v_			*D*_v_ × 10^4^		
*v*	Exp.^a^	Calc.^b^	Δ^c^	Exp.	Calc.	Δ	Exp.	Calc.	Δ
0	0.000	0.00	0.00	5.73651	5.7422	0.0057	3.5431	3.560	0.017
1	1431.978	1431.76	–0.22	5.55529	5.5608	0.0056	3.5569	3.573	0.016
2	2800.678	2800.37	–0.31	5.36751	5.3730	0.0055	3.5928	3.616	0.023
3	4102.330	4102.12	–0.21	5.16973	5.1753	0.0056	3.6753	3.704	0.029
4	5331.389	5331.51	0.12	4.95654	4.9623	0.0057	3.8273	3.868	0.041
5	6479.656	6480.36	0.70	4.71964	4.7254	0.0058	4.0954	4.161	0.066
6	7534.814	7536.34	1.52	4.44431	4.4504	0.0061	4.5742	4.694	0.120
7	8478.000	8480.34	2.34	4.10720	4.1116	0.0044	5.8007	5.710	–0.091
8	9279.653	9282.06	2.40	3.65877	3.6609	0.0022	7.3349	7.800	0.465
9	9892.724	9893.30	0.58	3.00781	2.9990	–0.0088	13.052	12.92	–0.13
10	10,249.407	10,244.46	–4.95	1.96873	1.9466	–0.0221	27.642	26.55	–1.10
11	10,352.250	10,347.82	–4.43	0.88390	0.9103	0.0264	45.349	42.02	–3.33

a The empirical band constants from ref (3).

b The values calculated using the adiabatic potential energy function; the zero-point energy is 738.96 cm^–1^.

c A difference between the calculated and experimental values.

The effects beyond the adiabatic approximation^36−39^ were investigated by applying second-order perturbational corrections to the vibrational and rotational terms of the effective vibration–rotation Hamiltonian of a diatomic molecule. The reduced nuclear mass μ in the vibrational term was replaced by the effective vibrational reduced mass μ_v_ defined as μ_v_ = μ/(1 + β) whereas that in the rotational term was replaced by the effective rotational reduced mass μ_r_ defined as μ_r_ = μ/(1 + α). The parameters β and α as a function of the internuclear distance *r* are given as^37,40^
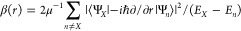
1and
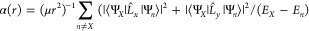
2Ψ_*X*_ and Ψ_*n*_ are the Born–Oppenheimer electronic wave functions of the ground and excited states, respectively, calculated at various *r*. *E*_*X*_ and *E*_*n*_ are the corresponding electronic total energies.  and  are the total angular momentum operators (for the Mg and H nuclei located at the *z* axis). The parameters β and α are related to the electronic contributions to the vibrational and rotational *g*-factors: *g*_v_^el^ and *g*_r_^el^, respectively. These relations are β = (*m*_e_/*m*_p_)*g*_v_^el^ and α = (*m*_e_/*m*_p_)*g*_r_^el^, where *m*_e_/*m*_p_ is the electron–proton mass ratio. The vibrational and rotational *g*-factors for the main isotopologue ^24^MgH were calculated at various internuclear distances using the CASSCF method with the aug-cc-pV5Z basis set and the extended active space consisting of 14 molecular orbitals (described above). For general theoretical details of such calculations, the reader is referred to refs (40–42). The calculations were performed using the DALTON package of ab initio programs.^43^

**Figure 6:**
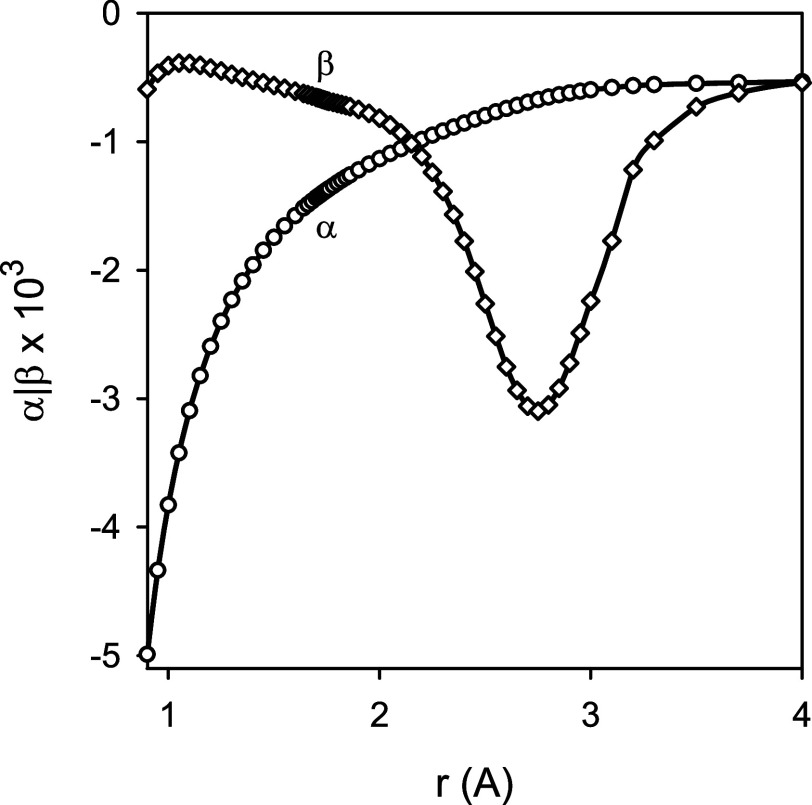
Nonadiabatic parameters α (circles) and β (diamonds) for the *X*^2^Σ^+^ state of ^24^MgH as a function of the internuclear distance *r*.

**Figure 7:**
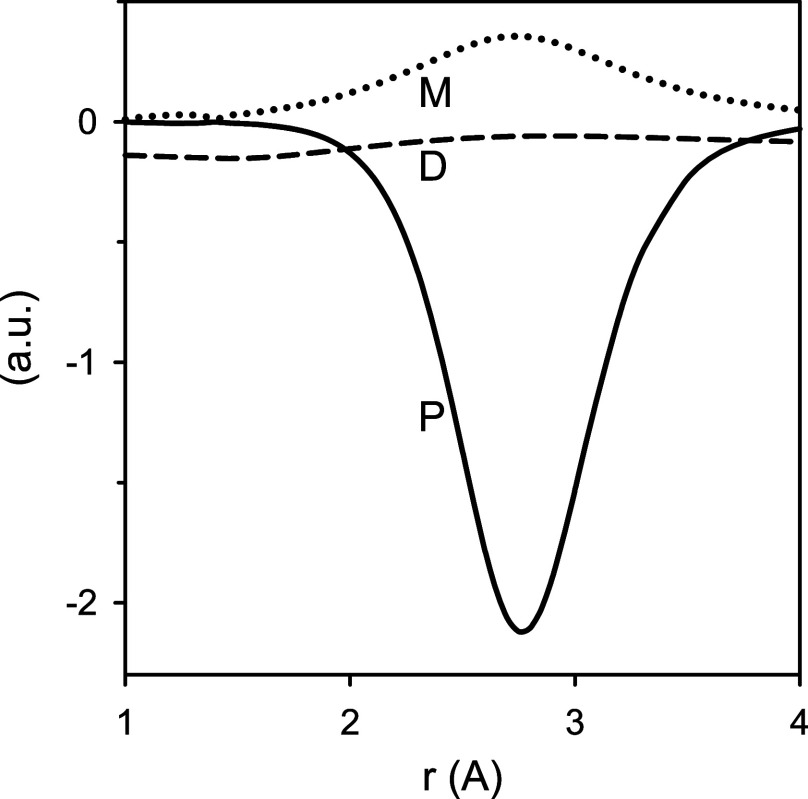
Perturbational term *P* (solid line) and its components, *M* and *D* (dotted and dashed lines, respectively; see the text), arising from the nonadiabatic interaction between the *X*^2^Σ^+^ and *B*′^2^Σ^+^ states of ^24^MgH, as a function of the internuclear distance *r*.

In the vicinity of the equilibrium configuration of ^24^MgH, the nonadiabatic parameters β and α were calculated to be about −0.67 × 10^–3^ and −1.40 × 10^–3^, respectively. Changes in the parameters β and α with the internuclear distance *r* are illustrated in Figure 6. The dependence of the parameter α on *r* is close to 1/*r*^2^, as might be expected from eq 2. This indirectly implies that the sum over excited electronic states, including matrix elements of the operators  and  and excitation energies, is nearly constant over a wide range about the ground-state equilibrium configuration of MgH. On a similar basis, see eq 1, the parameter β might be expected to vary only insignificantly with *r*. Somewhat surprisingly, this was found not to be the case for the ground electronic state of MgH. As shown in Figure 6, the parameter β (its absolute value) increases substantially with the increasing internuclear distance, reaching an extremum at *r* ≈ 2.7 Å. Then, the parameter β decreases uniformly, reaching asymptotically the value of −(*m*_e_/*m*_p_)*g*^nu^. The term *g*^nu^ is the (constant) nuclear contribution to the vibrational and rotational *g*-factors. Therefore, it was interesting to analyze individual second-order perturbational contributions to the parameter β. The transition matrix elements between the ground and excited electronic states of MgH were determined using the finite-difference three-point algorithm as implemented in the MOLPRO package of ab initio programs.^23,44^ The CASSCF/MRCI wave functions and energies for eight lowest doublet states of Σ^+^ symmetry were calculated using the aug-cc-pV5Z basis set and the extended active space described above. In the vicinity of the equilibrium configuration of MgH, the largest contributions to the parameter β arise from the first-to-third and fifth excited ^2^Σ^+^ electronic states. Contributions from the other excited ^2^Σ^+^ electronic states are by at least the order of magnitude smaller. However, at internuclear distances of about 2.7 Å, the contribution from the first excited state, *B*′^2^Σ^+^, predominates; it amounts to 98% of the parameter β value. Following eq 1, this contribution arises from the perturbational term *P* = *M*^2^/(*E*_*X*_ – *E*_*B*_), where M = |⟨Ψ_*X*_| – iℏ∂/∂*r*|Ψ_*B*_⟩| is calculated with the electronic wave functions Ψ of the *X*^2^Σ^+^ and *B*′^2^Σ^+^ states and *E*_*X*_ and *E*_*B*_ are the corresponding electronic total energies. Figure 7 illustrates the perturbational term *P* and its components, *M* and *D* = *E*_*X*_ – *E*_*B*_, as a function of the internuclear distance *r*. The difference of the electronic total energies *D* is fairly constant along the distance range shown. The shape of the perturbational term *P* as a function of *r* is thus determined by the square of the transition matrix element *M*. Consequently, a comparison of Figures 6 and 7 indicates that the shape of the nonadiabatic parameter β as a function of *r* is largely determined by the interaction between the *X*^2^Σ^+^ and *B*′^2^Σ^+^ states of MgH. Note that the location of an extremum of β as a function of *r* is fairly close to a minimum of the potential energy function for the *B*′^2^Σ^+^ state of MgH, being derived from the emission spectra of MgH to occur at *r* = 2.5940 Å.^45^

Changes in the vibrational term values *G*_v_ of ^24^MgH upon accounting for the nonadiabatic parameter β are given in Table 4, the column headed “VRM”. The changes Δ*G*_v_^na^ are calculated relative to the adiabatic vibrational term values listed in Table 3. These changes were also estimated by investigating the nonadiabatic interaction only between the *X*^2^Σ^+^ and *B*′^2^Σ^+^ states of MgH. In this case, the sum over excited electronic states defining the parameter β was truncated to the first term, i.e., the perturbational term *P* discussed above. Changes in the vibrational term values predicted in this way are listed in the column headed “TSO”. Last, the vibrational term values *G*_v_ of ^24^MgH were determined using the reduced mass μ calculated with the atomic masses of magnesium and hydrogen, μ_at_. In a spirit of the adiabatic approximation, the use of atomic (instead of nuclear) masses means that all electrons pertinent to a given atom follow very closely its nucleus during vibration. In terms of the nonadiabatic interaction, this corresponds to the parameter β being kept fixed at −(*m*_e_/*m*_p_)*g*^nu^. Changes in the vibrational term values predicted in this way are given in the column headed “ARM”. The changes Δ*G*_v_^na^ were found to be quite sizable, decreasing the vibrational term values of ^24^MgH by as much as 3 cm^–1^. As expected, the changes VRM and TSO look very much alike, concerning both the magnitude and the dependence on the vibrational quantum number *v*. Note that this dependence reflects the shape of the nonadiabatic parameter β as a function of the internuclear distance *r*; see Figures 6 and 7. As shown in Figure 4, the outer classical turning points for the highly excited vibrational levels *v* = 7–11 of ^24^MgH lie beyond the location of an extremum of β. All the changes Δ*G*_v_^na^ were predicted to be similar for low-lying vibrational levels. However, for the highly excited vibrational levels, the changes VRM and TSO were found to be larger (by a factor of 2) than the changes ARM.

**Table 4:** Changes in the Vibrational Term Values (Δ*G*_v_^na^, in Inverse Centimeters) for the *X*^2^Σ^+^ State of ^24^MgH Calculated Taking into Account the Nonadiabatic Effects

*v*	VRM^a^	TSO^b^	ARM^c^
0	–0.25	–0.22	–0.20
1	–0.72	–0.63	–0.56
2	–1.19	–1.03	–0.89
3	–1.64	–1.43	–1.18
4	–2.10	–1.84	–1.43
5	–2.56	–2.26	–1.62
6	–2.98	–2.66	–1.74
7	–3.30	–2.97	–1.76
8	–3.37	–3.06	–1.62
9	–2.83	–2.60	–1.25
10	–1.32	–1.23	–0.57
11	–0.29	–0.27	–0.13

a Predicted using the effective vibrational reduced mass μ_v_ = μ/(1 + β).

b Predicted using the parameter β modified to account only for the interaction between two states *X*^2^Σ^+^ and *B*′^2^Σ^+^; see the text.

c Predicted using the atomic reduced mass μ_at_.

**Figure 8:**
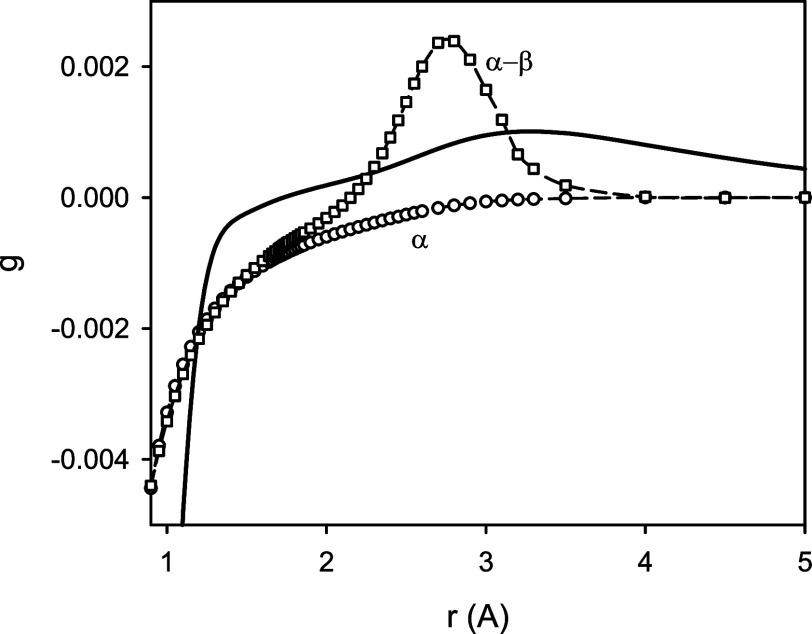
Experimental^4^ radial correction function *g*(*r*) for the nonadiabatic effects of ^24^MgH (solid line), in comparison to the predicted nonadiabatic functions α(*r*) (circles) and [α(*r*) – β(*r*)] (squares); see the text.

In analyses of the infrared spectra of MgH,^2−4^ the effective Hamiltonian of a diatomic molecule was applied. Following the theoretical work of Watson,^38,46^ the nonadiabatic terms associated with the kinetic energy operator were incorporated into the rotational part of the potential energy operator as the effective centrifugal-potential correction function [1 + *g*(*r*)].^47^ In general, the nonadiabatic correction *g*(*r*) can be expressed in terms of the nonadiabatic parameters β(*r*) and α(*r*) discussed above. The reduced mass μ was taken as the (constant) atomic reduced mass μ_at_ and, in the zeroth-order approximation, the correction *g*(*r*) was identical to α(*r*). In the first-order approximation, the correction *g*(*r*) became [α(*r*) – β(*r*)]; see eq 7 of ref (47). The shape of the correction function *g*(*r*) derived by Henderson et al.^4^ from the infrared spectra of ^24^MgH is shown in Figure 8 and compared with the functions α(*r*) and [α(*r*) – β(*r*)] predicted in this work.

The vibrational term values *G*_v_ and the effective rotational constants *B*_v_ and quartic centrifugal distortion constants *D*_v_ calculated for ^24^MgH taking into account the nonadiabatic effects are given in Table 5. Except for the *v* = 10 and 11 vibrational levels, the experimental vibrational term values of ^24^MgH are reproduced to within 1.4 cm^–1^, whereas the effective rotational constants are reproduced to within 0.0029 cm^–1^ (the root-mean-square deviation). The corresponding accuracy limits for these spectroscopic constants obtained within the adiabatic approximation, see Table 3, are 1.3 and 0.0057 cm^–1^, respectively. Differences between the calculated and experimental vibrational term values Δ*G*_v_ of ^24^MgH are illustrated in Figure 5. The remaining errors in the predicted spectroscopic constants of ^24^MgH are likely due to approximations inherent to the MR-ACPF method.

**Table 5:** Vibrational Term Values (*G*_v_) and the Effective Rotational (*B*_v_) and Quartic Centrifugal Distortion (*D*_v_) Constants (All in Inverse Centimeters) for the *X*^2^Σ^+^ State of ^24^MgH

	*G*_v_			*B*_v_			*D*_v_ × 10^4^		
*v*	Exp.^a^	Calc.^b^	Δ^c^	Exp.	Calc.	Δ	Exp.	Calc.	Δ
0	0.000	0.00	0.00	5.73651	5.7341	–0.0024	3.5431	3.543	–0.001
1	1431.978	1431.28	–0.69	5.55529	5.5530	–0.0023	3.5569	3.555	–0.002
2	2800.678	2799.43	–1.25	5.36751	5.3655	–0.0020	3.5928	3.598	0.005
3	4102.330	4100.72	–1.61	5.16973	5.1681	–0.0016	3.6753	3.686	0.011
4	5331.389	5329.65	–1.74	4.95654	4.9554	–0.0011	3.8273	3.849	0.021
5	6479.656	6478.05	–1.61	4.71964	4.7190	–0.0006	4.0954	4.141	0.045
6	7534.814	7533.60	–1.21	4.44431	4.4446	0.0003	4.5742	4.670	0.095
7	8478.000	8477.28	–0.72	4.10720	4.1068	–0.0004	5.8007	5.677	–0.124
8	9279.653	9278.93	–0.72	3.65877	3.6579	–0.0009	7.3349	7.748	0.413
9	9892.724	9890.72	–2.01	3.00781	2.9997	–0.0081	13.052	12.81	–0.24
10	10,249.407	10,243.39	–6.02	1.96873	1.9529	–0.0158	27.644	26.36	–1.28
11	10,352.250	10,347.78	–4.47	0.88390	0.9157	0.0318	45.349	41.68	–3.67

a The empirical band constants from ref (3).

b The values predicted taking into account the nonadiabatic effects; the zero-point energy is 738.72 cm^–1^.

c A difference between the calculated and experimental values.

The dissociation energy *D*_0_ of the main isotopologue ^24^MgH in its *X*^2^Σ^+^ state is determined in this work to be 10,362 cm^–1^, compared with the experimental value^4^ of 10,365.14 cm^–1^. The vibrationally averaged internuclear distance ⟨*r*⟩ is calculated for the ground vibrational state to be 1.7530 Å. The vibrationally averaged rotational and vibrational *g*-factors are predicted to be ⟨*g*_r_⟩ = −1.578 and ⟨*g*_v_⟩ = −0.248, respectively.

To summarize, descriptors of the molecular dynamics and structure—the vibrational fundamental wavenumbers ν and the effective ground-state rotational constants *B*_0_—for the *X*^2^Σ^+^ state of the most-abundant MgH isotopologues are given in Table 6. The values predicted in this study using the adiabatic and nonadiabatic approaches are compared with those obtained by Henderson et al.^4^ from the experimentally adjusted Morse/Long-Range potential energy function of MgH and the Born–Oppenheimer breakdown functions.

**Table 6:** Vibrational Fundamental Wavenumbers (ν) and the Effective Ground-State Rotational Constants (*B*_0_, All in Inverse Centimeters) for the *X*^2^Σ^+^ State of Various MgH Isotopologues

	ν			*B*_0_		
isotopologue	Exp.^a^	Calc.^b^	Δ^c^	Exp.	Calc.	Δ
Adiabatic						
^24^MgH	1431.978	1431.76	–0.22	5.73651	5.7422	0.0057
^24^MgD	1045.844	1045.50	–0.34	3.00095	3.0019	0.0010
^24^MgT	875.481	875.15	–0.33	2.08558	2.0858	0.0002
^25^MgH	1430.871	1430.65	–0.22	5.72732	5.7330	0.0057
^26^MgH	1429.851	1429.63	–0.22	5.71888	5.7246	0.0057
Nonadiabatic						
^24^MgH	1431.978	1431.28	–0.69	5.73651	5.7341	–0.0024
^24^MgD	1045.844	1045.33	–0.51	3.00095	2.9997	–0.0012
^24^MgT	875.481	875.05	–0.43	2.08558	2.0847	–0.0008
^25^MgH	1430.871	1430.18	–0.69	5.72732	5.7249	–0.0024
^26^MgH	1429.851	1429.16	–0.69	5.71888	5.7165	–0.0024

a The experimental spectroscopic constants from ref (4).

b The values predicted using the adiabatic and nonadiabatic approaches.

c A difference between the calculated and experimental values.

The electric dipole moment of MgH in its *X*^2^Σ^+^ state was determined as an expectation value for the electronic wave function calculated at the MR-ACPF/aug-cc-pCV8Z(*i*) level of theory, with the extended active space described above. The predicted dipole moment as a function of the internuclear distance is given in Table S2 of the Supporting Information The vibrationally averaged electric dipole moment of ^24^MgH was predicted for the ground vibrational state to be 1.386 D. The natural-bond-orbital atomic net charges in the *X*^2^Σ^+^ state of MgH were determined at the MR-ACPF/aug-cc-pCVQZ level of theory to be 0.73*e* and −0.73*e* on the magnesium and hydrogen atoms, respectively. To our knowledge, the electric dipole moment of MgH was not determined experimentally so far.

## Conclusions

In conclusion, the accurate potential energy function of magnesium monhydride in the ground electronic state *X*^2^Σ^+^ was determined in the state-of-the-art ab initio calculations. The vibration–rotation energy levels of the main isotopologue ^24^MgH were predicted to near the “spectroscopic” accuracy. The scalar relativistic, adiabatic, and nonadiabatic effects were taken into account. For the vibrational energy levels up to *v* = 9, the remaining differences between the experimentally observed and theoretically predicted spectroscopic constants of ^24^MgH are likely due to approximations inherent to the MR-ACPF method, applied in this work to determine the electronic wave function and properties. The analogous differences for *v* = 10 and 11 clearly do not follow that accuracy pattern. It is an open question whether this discrepancy results also from the theoretical method applied or from a misassignment of the experimental data. The nonadiabatic interaction between the *X*^2^Σ^+^ and *B*′^2^Σ^+^ states of MgH should be explicitly accounted for in a future analysis of the high-resolution vibration–rotation spectra.

## Supporting Information Available

The predicted Born–Oppenheimer (CV + R) and adiabatic (CV + R + D) potential energy functions for ^24^MgH in its *X*^2^Σ^+^ state and electric dipole moment of MgH as a function of the internuclear distance (PDF)
