# Supplementary material for: Ab Initio Ground-State Potential Energy Function and Vibration–Rotation Energy Levels of Magnesium Monohydride
Source: J Phys Chem A. 2024 May 1;128(19):3855–63. doi: 10.1021/acs.jpca.4c01757 (PMC11103694; doi:10.1021/acs.jpca.4c01757)
Supplement: Supplementary file 1 — jp4c01757_si_001.pdf [file jp4c01757_si_001.pdf]

Ab Initio Ground-State Potential Energy Function and Vibration-Rotation Energy Levels of Magnesium Monohydride

Jacek Koput
Department of Chemistry, Adam Mickiewicz University, 61-614 Poznan, Poland

Table S1: The predicted Born-Oppenheimer (CV+R) and adiabatic (CV+R+D) potential energy functions for ^{24}MgH in its X $^2\Sigma^+$ state

r (Å)	CV+R (cm^{-1})	CV+R+D (cm^{-1})
0.90	66830.7	66890.5
0.95	52051.3	52104.9
1.00	39693.4	39741.9
1.05	29409.3	29453.7
1.10	20893.9	20934.6
1.15	13881.2	13918.7
1.20	8140.8	8175.4
1.25	3475.2	3507.1
1.30	-284.3	-255.0
1.35	-3281.1	-3254.1
1.40	-5636.7	-5611.8
1.45	-7454.1	-7431.2
1.50	-8820.2	-8799.2
1.55	-9808.9	-9789.6
1.60	-10482.4	-10464.7
1.64	-10830.0	-10813.5
1.66	-10948.3	-10932.4
1.68	-11033.2	-11017.8
1.70	-11087.0	-11072.2
1.71	-11103.1	-11088.4
1.72	-11112.2	-11097.8
1.73	-11114.7	-11100.6
1.74	-11110.9	-11097.0
1.75	-11100.9	-11087.3
1.76	-11085.1	-11071.7
1.78	-11036.7	-11023.8
1.80	-10967.6	-10955.1
1.82	-10879.4	-10867.3

1.84	-10773.6	-10761.9
1.86	-10651.8	-10640.5
1.90	-10365.6	-10355.0
1.95	-9940.7	-9930.9
2.00	-9457.4	-9448.3
2.05	-8930.5	-8922.1
2.10	-8373.3	-8365.4
2.15	-7796.9	-7789.4
2.20	-7211.3	-7204.1
2.25	-6625.3	-6618.2
2.30	-6046.4	-6039.3
2.35	-5481.4	-5474.3
2.40	-4936.2	-4928.9
2.45	-4416.0	-4408.3
2.50	-3924.9	-3917.0
2.55	-3466.4	-3458.2
2.60	-3043.1	-3034.5
2.65	-2655.6	-2646.8
2.70	-2307.2	-2298.4
2.75	-1995.1	-1986.5
2.80	-1718.5	-1710.4
2.85	-1476.4	-1468.8
2.90	-1264.8	-1257.7
2.95	-1083.1	-1076.8
3.00	-927.4	-921.8
3.10	-681.8	-677.6
3.20	-505.7	-502.7
3.30	-380.4	-378.3
3.50	-227.9	-226.9
3.70	-148.9	-148.5
4.00	-91.2	-91.1
4.50	-45.8	-45.8
5.00	-24.7	-24.7
6.00	-8.4	-8.4
8.00	-1.4	-1.4
12.0	-0.1	-0.1
20.0	0.0	0.0

50.0	0.0	0.0

Table S2: The predicted electric dipole moment (μ) for MgH in its X $^2\Sigma^+$ state

r (Å)	μ (a.u.)
0.90	0.142398
0.95	0.121126
1.00	0.096465
1.05	0.068472
1.10	0.037320
1.15	0.003241
1.20	-0.033516
1.25	-0.072698
1.30	-0.114057
1.35	-0.157354
1.40	-0.202353
1.45	-0.248819
1.50	-0.296513
1.55	-0.345186
1.60	-0.394577
1.64	-0.434418
1.66	-0.454396
1.68	-0.474386
1.70	-0.494367
1.71	-0.504348
1.72	-0.514318
1.73	-0.524273
1.74	-0.534212
1.75	-0.544133
1.76	-0.554032
1.78	-0.573750
1.80	-0.593341
1.82	-0.612779
1.84	-0.632035
1.86	-0.651081
1.90	-0.688425
1.95	-0.733364

2.00	-0.775859
2.05	-0.815309
2.10	-0.851050
2.15	-0.882370
2.20	-0.908514
2.25	-0.928692
2.30	-0.942110
2.35	-0.948010
2.40	-0.945725
2.45	-0.934750
2.50	-0.914825
2.55	-0.885997
2.60	-0.848728
2.65	-0.803886
2.70	-0.752762
2.75	-0.696977
2.80	-0.638345
2.85	-0.578697
2.90	-0.515627
2.95	-0.459342
3.00	-0.406371
3.10	-0.312855
3.20	-0.237150
3.30	-0.178079
3.50	-0.099220
3.70	-0.055248
4.00	-0.023318
4.50	-0.003229
5.00	-0.000938
6.00	-0.000384
8.00	-0.000065
12.0	-0.000003
20.0	0.000000
